# Challenges and opportunities for implementing evidence-based antenatal care in Mozambique: a qualitative study

**DOI:** 10.1186/s12884-015-0625-x

**Published:** 2015-09-02

**Authors:** Adriano Biza, Ingeborg Jille-Traas, Mercedes Colomar, Maria Belizan, Jennifer Requejo Harris, Beatrice Crahay, Mario Merialdi, My Huong Nguyen, Fernando Althabe, Alicia Aleman, Eduardo Bergel, Alicia Carbonell, Leonardo Chavane, Therese Delvaux, Diederike Geelhoed, Metin Gülmezoglu, Celsa Regina Malapende, Armando Melo, Nafissa Bique Osman, Mariana Widmer, Marleen Temmerman, Ana Pilar Betrán

**Affiliations:** International Center for Reproductive Health (ICRH-Mozambique), Maputo, Mozambique; Montevideo Clinical and Epidemiological Research Unit (UNICEM), Montevideo, Uruguay; Institute for Clinical Effectiveness and Health Policy (IECS), Buenos Aires, Argentina; Institute for International Programs, Johns Hopkins Bloomberg School of Public Health, Baltimore, MD USA; Department of Reproductive Health and Research, UNDP-UNFPA-UNICEF-WHO-World Bank Special Programme of Research, Development and Research Training in Human Reproduction, World Health Organization, Avenue Appia 20, Geneva, CH-1211 Switzerland; Mozambique Country Office, World Health Organization, Maputo, Mozambique; Mozambique Ministry of Health, Maputo, Mozambique; Institute of Tropical Medicine, Antwerp, Belgium; Central de Medicamentos e Artigos Médicos, Maputo, Mozambique

## Abstract

**Background:**

Maternal mortality remains a daunting problem in Mozambique and many other low-resource countries. High quality antenatal care (ANC) services can improve maternal and newborn health outcomes and increase the likelihood that women will seek skilled delivery care. This study explores the factors influencing provider uptake of the recommended package of ANC interventions in Mozambique.

**Methods:**

This study used qualitative research methods including key informant interviews with stakeholders from the health sector and a total of five focus group discussions with women with experience with ANC or women from the community. Study participants were selected from three health centers located in Maputo city, Tete, and Cabo Delgado provinces in Mozambique. Staff responsible for the medicines/supply chain at national, provincial and district level were interviewed. A check list was implemented to confirm the availability of the supplies required for ANC. Deductive content analysis was conducted.

**Results:**

Three main groups of factors were identified that hinder the implementation of the ANC package in the study setting: a) *system or organizational*: include chronic supply chain deficiencies, failures in the continuing education system, lack of regular audits and supervision, absence of an efficient patient record system and poor environmental conditions at the health center; b) *health care provider factors*: such as limited awareness of current clinical guidelines and a resistant attitude to adopting new recommendations; and c) *Users*: challenges with accessing ANC, poor recognition amongst women about the purpose and importance of the specific interventions provided through ANC, and widespread perception of an unfriendly environment at the health center.

**Conclusions:**

The ANC package in Mozambique is not being fully implemented in the three study facilities, and a major barrier is poor functioning of the supply chain system. Recommendations for improving the implementation of antenatal interventions include ensuring clinical protocols based on the ANC model. Increasing the community understanding of the importance of ANC would improve demand for high quality ANC services. The supply chain functioning could be strengthened through the introduction of a kit system with all the necessary supplies for ANC and a simple monitoring system to track the stock levels is recommended.

**Electronic supplementary material:**

The online version of this article (doi:10.1186/s12884-015-0625-x) contains supplementary material, which is available to authorized users.

## Background

Maternal mortality remains a major problem in many resource-poor settings. Estimates show that approximately 289,000 maternal deaths occurred in 2013, 99 % in developing countries. Although this estimate represents a decline of 45 % from 1990 levels, 40 countries, including Mozambique, are still experiencing high levels of maternal mortality (maternal mortality ratio equal to or greater than 300 maternal deaths per 100,000 live births). Mozambique’s maternal mortality ratio was 480 per 100,000 live births in 2013, indicating that urgent efforts are needed if the country is to achieve its Millennium Development Goal 5a target of 258 maternal deaths per 100,000 live births by 2015 and expand access to needed services for all women post-2015 [1].

Antenatal care (ANC) is an important determinant of safe delivery [2] and represents a key opportunity for reaching pregnant women with services that can improve their health and the health of their unborn baby [3]. ANC can reduce maternal and perinatal morbidity and mortality directly through the detection and treatment of pregnancy-related illnesses, and indirectly through the identification of women at increased risk of delivery complications [4, 5]. Associations found between ANC utilization and skilled attendance at childbirth also suggest that ANC can improve obstetric outcomes through promotion of skilled delivery care and counseling on birth planning and complication readiness [2, 6, 7].

In December 2008, the Ministry of Health of Mozambique launched the *Integrated Plan for the Achievement of Millennium Development Goals (MDGs) 4 and 5*. The plan included an integrated package of interventions for pregnant women, newborns and children under 5 years of age. The interventions recommended for pregnant women are based on the World Health Organization’s (WHO) ANC model which includes the delivery of services scientifically proven to improve maternal, perinatal and neonatal outcomes [8, 9]. Table 1 shows the elements included in each ANC visit in the context of the ANC package of the Ministry of Health in Mozambique.

**Table 1:** Elements included in each ANC visit in the context of the ANC package

Elements of ANC	1^st^ ANC	2^nd^ ANC	3^rd^ ANC	4^th^ ANC and following
1. Clinical physical examination	X	X	X	X
2. Obstetric examination	X	X	X	X
3. Measurement of haemoglobin level	X		From 32 weeks	
4. Assessing proteinuria	X	X	X	X
5. Measurement of blood pressure	X	X	X	X
6. Performance of syphilis test and treatment	X			
7. Prevention of anaemia and deworming				
7.1 Deworming (mebendazole)	X			
7.2 Ferrous sulfate + folic acid	X	X	X	X
8. Prevention of malaria				
8.1 ITP (Sulfadoxine-pyrimethamine)	X	X	X	X
8.2 Mosquito net	X			
9. HIV testing and counselling	X			X
10. Vaccination	X	X	X	
11. Complementary intervention: Provision of ARV treatment	X	X	X	X

A meeting of experts was convened in Maputo in 2009 by the Ministry of Health, the World Health Organization (WHO), the International Centre for Reproductive Health (ICRH), and the Flanders International Cooperation Agency (FICA) to discuss the country’s early experiences with implementation of the ANC model. Health system deficiencies and lack of motivation and knowledge among care providers were identified as potential determinants of slow uptake of the ANC model.

To further explore these and other underlying factors responsible for the slow roll-out of the ANC model, the WHO, the Ministry of Health of Mozambique and the International Center for Reproductive Health – Mozambique (ICRH-M) launched a two-pronged study in 2010. The formative research component of the study, described in this paper, assessed the views of pregnant women attending ANC and women who had been pregnant recently as well as the views of health care provider on available ANC services, and supply chain factors hindering the implementation of the ANC model. The findings of this study were used to inform the development of an intervention to improve delivery of antenatal care to be assessed through a subsequent randomized controlled trial [10].

## Methods

### Study setting

This was a cross-sectional study conducted between May and July 2011 in three health centers located in the provinces of Maputo city, Tete, and Cabo Delgado, each representing a distinct geographical region in Mozambique (south, central, and north). These health centers were purposively selected from among the 10 centers selected by the Ministry of Health for inclusion in the randomized trial. Among the 10 facilities in the trial, these three health centers, Montepuez (North), Matola (South) and Songo (Central) were the third, fifth and tenth in terms of the number of first ANC visits with 4182, 3276 and 1281 visits in 2011 (See Additional file 1). At the time of the study, none of the three facilities were fully implementing the WHO ANC model adapted for the Mozambique context (henceforth called the "ANC model"). Table 1 shows the components of the ANC model.

### Data collection methods

To best gain an understanding of the barriers that hinder practitioners from delivering the evidence-based practices listed in the ANC model and women’s underutilization of services in the study setting, qualitative methods were used. These included focus group discussions and semi-structured interviews designed for each type of informant. Table 2 presents information on the number of interviews and focus group discussions per province and from Maputo (the Ministry of Health at National level). A structured checklist to confirm the availability of supplies needed to provide all of the services included in the ANC model was also used. Participants in the focus groups and interviews did not consent recording of the discussions.

**Table 2:** Health sector participant category and number of sessions held

Category	Maputo City	Tete Province	Cabo Delgado Province	Central MoH
	Number of interviews			
Health sector officer at national level (including officers for MCH)	NA	NA	NA	5
Health sector officer at provincial level (including officers for MCH)	5	4	4	NA
Health sector officer at district level (including officers for MCH)	6	3	4	NA
NGO representative	1	3	3	5
Supply Chain responsible (all three levels)	3	3	2	4
Facility or Program responsible	2	2	2	NA
MCH nurse	2	2	2	NA
Laboratory (responsible and technical staff)	1	2	2	NA
	Number of focus groups (number of participants)			
Pregnant women users of ANC care	1 (8)	1 (10)	1 (7)	NA
Women from the community	-	1 (13)	1 (6)	NA

*NA* Not applicable, *MoH* Ministry of Health

#### Focus groups

Three focus groups were conducted with women attending ANC at the time of the study, and two were conducted with women living in surrounding communities who had a recent experience with ANC and delivery services. Women attending ANC at the time of the study were recruited if they had attend at least one ANC visit at the study facilities. Health facility staff assisted in identifying potential study participants until the quota of participants for each focus group was reached. In the surrounding communities, women were identified with the help of community leaders and the permission of district authorities. Focus groups lasted approximately 90 minutes and were held in locations convenient to the participants. Snacks and beverages were offered to participants.

All focus group discussions involved 7 to 13 women and were conducted by a team including a trained facilitator fluent in Portuguese and English, an observer responsible for taking notes (IJT or AB), and a translator of other local languages or dialects to help with communication. Facilitators were selected using the educational level criteria combined with experience in data collection methods through in-depth individual interviews and conducting focus discussions, or experience in community animation (See Additional file 1 for more detailed information on the focus groups and facilitators).

#### Interviews

Semi structured interviews were conducted with the following key informants: (i) health sector senior officers at national level in charge of policy and guideline development for maternal, newborn and child health (MNCH) including all aspects of care related to the ANC model; (ii) health sector senior officers at provincial level responsible for providing guidance on the content of MNCH strategies and for monitoring the implementation of health programs at provincial level; (iii) health sector senior officers at district level in charge of supervising the quality of MNCH services including the supply chain management; (iv) Non Governmental Organizations (NGO) representatives engaged in supporting the delivery of maternal and child health (MCH) services and/or supply chain management; (v) staff at national, provincial and district level responsible for supply chain management of medicines and other health commodities needed for ANC services; (vi) MCH nurses responsible for delivering ANC services; and (vii) in each of the selected health centers, staff involved in supply chain management of the medicines and other materials necessary for full implementation of the ANC model.

The interviews were carried out by two investigators (IJT and AB) in locations convenient to the interviewee, and lasted approximately 1 hour. Notes were taken during focus groups and interviews, translated into English, and written up in Microsoft word.

#### Structured check list

A checklist was used to assess the availability of equipment, medicines and other health commodities needed for full implementation of the ANC package. It was filled out during interviews with responsible staff in ANC consultation rooms, pharmacies, laboratories and storage rooms at the health center level, and by taking an inventory of stocks available in warehouses at the central, provincial, and district level. Registers were checked for information on stock-outs in the past. Two investigators (IJT, AB) worked closely with the staff from the Ministry of Health involved in supply chain management and logistics to examine the stocks in the three selected health centers, as well as central, provincial and district warehouses in order to follow the supply chain from when products arrive in Maputo (central level) to when they reach the health facilities via the provincial and district warehouses.

The study protocol was approved by the Research Project Review Panel of the UNDP/UNFPA/UNICEF/WHO/World Bank Special Programme of Research, Development and Research Training in Human Reproduction at the Department of Reproductive Health and Research of WHO, and the WHO Research Ethics Review Committee, Geneva, Switzerland. The *Comité Nacional de Bioética para a Saúde* of the Ministry of Health in Mozambique also approved the study. All participants gave their written informed consent.

### Data analysis

The guides for the focus groups and interviews were developed based on the study objectives and from other guides used in similar studies that were adapted to the Mozambique context. A deductive content analysis was conducted [11]. This type of analysis consist on classifying the elements of a message in order to arrive to the comprehension of the whole sense, guided by the existing knowledge [12]. As per protocol, the data analysis was performed simultaneously triangulating qualitative data (from discussion groups and individual interviews) with quantitative data (from check list).

The interviews and focus group transcripts were coded simultaneously according to themes and categories based on the interview, focus group questions and study objectives. Matrices were developed to facilitate comparisons across the transcripts material and to retain the context of the data (i.e. which focus group, characteristics of the interviewee) [13]. Finally abstraction and interpretation of data was performed. As part of this analysis, direct quotations representative of participants’ opinions were selected. Quotations were translated from Portuguese to English for the purpose of this article.

The material collected through the checklists was entered into an Excel spreadsheet and simple frequencies were calculated for each variable. To assure the validity of the research, check for consistency in findings across data sources and to capture different dimensions and perspectives of the same phenomenon, all data was triangulated. Triangulation was achieved by using different types of sources of information as well as considering and combining different methodologies, quantitative (use of checklists) and qualitative. The underlying principle of this technique lies in the notion that confidence in the results increases if different methods are used.

## Results

The findings identified a number of interrelated factors that may prevent or delay access to ANC, as well as factors that may facilitate its implementation. These factors were organized into three categories: a) system or organizational; b) health provider factors (i.e. knowledge, skills and attitudes); and c) demand-side factors related to women’s use of ANC.

### Health system and organizational factors

In this section, the system and organizational factors that may hinder the delivery of the ANC package are described. Table 3 shows a summary of these factors.

**Table 3:** Organizational or system and health center level factors for the Implementation of ANC model

Factor	Main barrier
*System or organizational factors*	
*Supply chain*	Fragmentation due to multiplicity of supply chains and sub-supply chains
	Lack of coordination between levels
	Lack of knowledge of the procurement and supply chain management concept
	Lack of qualified personnel with experience in supply management
	Lack of protocols
	Lack of reliable data for supply management
	Deficiencies on storage system
	Difficulties for the distribution due to transportation deficiencies
*Audit and Supervision*	Lack of regular supervision and monitoring visits affects the care provided
*Health care providers continuing education system*	Limited impact of the existing training models on ANC model
*Human Resources at health center level*	Staff shortage
	Staff turnover
	High workload
*Goods and products at health center level*	Lack of supplies
*Environment conditions at HC level*	Lack of appropriate infrastructure to conduct the interventions of the package in an integrated way
*Patient Data Record System at health center level*	Deficiencies on record system regarding patients’ information
	Absence of training on data collection
	Lack of data collection forms
	Data recording fragmentation and duplication
	Deficiencies on data analysis
*Factors related to the health providers knowledge, skills and attitudes*	
*Health providers knowledge and skills*	Lack of awareness on guidelines and protocols implementation
*Health care provider attitudes*	Resistance to follow the procedures

#### Supply chain

The ANC model requires availability of at least 42 different products at the health center during the first consultation (e.g. medicines, health commodities, equipment and instruments, laboratory reagents and tests). In the three study health centers, 10 different sub-supply chains provide these 42 products. None of the health centers were observed to have all of the required products available at the time of data collection.

The malfunctioning of the supply chain system is related to problems with planning, procurement, storage and distribution of goods. Barriers identified to effective planning and management include (i) fragmentation of the system due to multiplicity of chains and sub-chains; (ii) lack of coordination between each level of the supply chain (i.e. health center, district, provincial and national warehouses) due to communication problems; (iii) lack of supply chain management personnel qualified and experienced resulting in a lack of awareness of procedures; and (iv) lack of defined protocols and instructions for supply chain management.

Basic and general procedures are in place for supply chain management throughout the public health system. However they are not always followed for a variety of reasons. The current system of inventory and record keeping is poorly designed, making it a challenge for health center staff to be able to anticipate needs and to avoid stock outs. Supplies are often not re-ordered until after stock-outs have already occurred. The absence of adequate storage rooms and refrigeration at the health centers also creates problems with ensuring stock of sufficient supplies in appropriate conditions. Transportation services are not properly designated for the distribution of health sector supplies, mainly from the district level to the health centers. Consequently, ambulances, which often breakdown or are required for transport of patients, are unofficially used for this purpose.

Health care providers interviewed noted that supplies needed for the interventions are generally not easily accessible and that they spend lots of time trying to obtain them:

*"We do not measure the blood pressure because we do not have the device. We have many tensiometers but we do not have the stethoscopes. We only received sphygmomanometer (…) I'm here since February, and we haven´t assessed pregnant women’s blood pressure…" [Nurse at Health Center]*

#### Continuing education system

Those interviewed noted that problems with the continuing education system to help providers stay up-to-date on the latest evidence-based guidelines hinders provider ability to fully implement the ANC model.

The continuing education system was designed as a "training of trainers program". This program focuses on MCH district supervisors, this model is seen by the nurses as exclusionary; it does not cover most of the nurses who directly provide services to users. Those who attend the training sessions do not replicate the trainings at the health center level; they complain on not receiving additional benefits (per diem) for participating on the sessions. Participants highlighted that they don´t find the sessions applicable to their daily practice. Transfer of information from the central level is not conducted systematically leading to insecurity and doubts regarding current care recommendations among health providers. Clinical guidelines and protocol updates are not readily available at peripheral health facilities.

#### Audit and supervision

Health center informants identified the scarcity of supervision and monitoring visits as a barrier for full implementation of the ANC model. They indicated that the bi-annual supervision visits are not completed as planned. They perceive that increased supervision and training would be useful to refresh their skills, as well as for mentoring purposes and to help them address any difficulties they are facing.

### Human resources at health center level

Health care providers stated that they endure a heavy workload and feel overburdened. MCH nurses are expected to deliver many services - ANC, delivery care, postpartum check-ups, family planning, basic emergency obstetric care, and newborn care. This situation is exacerbated at peripheral health centers where there are a single MCH nurse on duty.

For ANC provision, nurses are being asked to provide an increasing number of services during the first visit, which is often the only visit women attend. The limited time assigned for each visit (less than 10 to 15 minutes) results in nurses choosing to provide specific services from the ANC package at the expense of establishing a good rapport with the women and providing adequate counselling (particularly important for prevention of mother to child transmission (PMTCT) of Human Immunodeficiency Virus (HIV) services and syphilis tests). There are also high absenteeism and staff turnover rates, which increases the volume of work for the few remaining skilled and experienced providers.

Nurses perceive that they are unfairly blamed when service goals are not reached, which contributes to a tense environment. They are concerned that the increase in their responsibilities to provide all of the services in the ANC model is not accompanied by either an increase in the number of health providers devoted to providing care or by a reduced patient load:

*"To provide the ANC package we experience great staff difficulties. Many clinics have only one nurse to provide antenatal care, so it is difficult to do everything with a desired quality (…) While the problem of human resources is not solved it will be difficult to improve the quality of the ANC provided". [Maternal Health Officer]*

#### Environmental conditions at health center level.

Women identified long waiting times and inadequate infrastructure as barriers to seeking ANC at the health centers. Women reported that they have to wait a long time for ANC due to the absence of an appointment system and shortage of human resources. Nurses typically work in the morning and all ANC consults, therefore, must occur in the morning hours. Women report lack of privacy of the exam rooms for counseling and other ANC services. These conditions also negatively impact the motivation of nurses to provide care.

#### Patient record system at health center level

Health care providers reported that data were not routinely collected at the health centers because they often run out of data collection tools due to chronic shortages of office supplies. From their perspective, they do not receive the training needed to guarantee good quality of data collection and analysis.

There is an absence of protocols for data analysis, for example, and for sharing clinical results with health care providers and other staff. Moreover, multiple reporting systems co-exist, leading to registration duplicates and inefficiencies in the quality control process.

### Factors related to the health care providers’ knowledge, skills, attitudes and behaviors

A summary of provider knowledge, skills, attitudes and behaviors that were identified as influencing successful implementation of the ANC model is presented in Table 3.

#### Knowledge and skills

Health care providers expressed a lack of awareness of current clinical guidelines and protocols (e.g. screening for HIV and syphilis or deworming protocols). Also, blood samples are often not adequately taken. Providers often take less blood than necessary and/or wait less time than indicated to obtain accurate results for HIV testing. Both situations might lead to invalid test results.

#### Attitudes, and behaviors

Health care providers are unable to deliver the full ANC package since they cannot establish a routine and effective workflow due to frequent stock outs of different supplies. Health care providers expressed that lack of time impairs their relationships with their clients. Even when the provider is aware of the existence of protocols, lack of time and adequate supervision impinges on their ability to fully implement them.

Authorities stated that health care providers are unmotivated and have a resistant attitude towards their responsibilities. Nurses contend that the circumstances they face in the health system result in negativity and a loss of motivation.

### Women’s experiences

Focus groups held with women in the community and at the health centers identified other important factors related to implementation of the ANC model, catalogued below as either internal (related to individual characteristics) or external factors (related to the environment, organization and rules of the health system) (Table 4). Most respondents experienced several challenges when seeking ANC at the health centers.

**Table 4:** Pregnant women’s barriers and facilitators for the use of ANC

Factor & components	Barrier	Facilitator
*Internal factors*		
*Knowledge and Beliefs: Perceptions on the ANC importance*	Not aware of each component value neither its significance, they are lay across beliefs.	It is recognized. Some women seem to be giving up the idea of receiving care at the community level
		They seek to "open the record" in order to make easier admission for delivery at health center
*Attitudes & Behaviors: Approach to the health system for ANC and delivery*	Arrival to ANC care during 2nd and 3rd trimester, due to impossibility to prove the pregnancy	Willingness to deliver at institutional level.
	Perception that they will not receive any care until pregnancy is evident.	
*External factors*		
*Accessibility: Transportation*	Distance and lack of transportation prevents accessing to health centers and laboratory	
*Accessibility: Economic*	Pregnancy test are not affordable, resulting into late consultation	
	Lack of integral free treatment to partners for syphilis treatment, prevent women to succeed in treatment	

#### Internal factors: Women’s knowledge and beliefs

Although supply stock outs, an unfriendly environment, and long waiting times discourage women from seeking ANC, women recognize the importance of ANC to check their own health status and their unborn baby’s health.

*"… It is important to know my health and also how the baby is. Sometimes I feel sick and I don´t know what's going on. Nowadays there are many diseases, I´m afraid to have them…" [Focus Group with ANC Users]*

When asked about the importance of each of the components of the ANC package, however, women were unable to respond. Women’s beliefs affected their adherence to some of the recommendations in the ANC model. For example, women’s adherence to the full vaccination plan is poor due to their belief that receiving the first dose of tetanus vaccine is sufficient for the wellbeing of the baby and that other doses are not so necessary.

Women acknowledged that they are more open to receiving ANC at the health center than previous generations, and perceive a benefit to receiving care from both the formal public health system and from relatives or traditional community midwives.

*"Most women come to the hospital now, those who do not want to come, trust on their relatives, others trust on the matrons working in neighborhoods" [FGD with ANC Users]*

Women are motivated to seek ANC since they perceive that accessing ANC will give them formal entrance into the health system. Both women and nurses interviewed recognize that the main motivation for seeking ANC at the health center is the so called “opening the card” during the first consultation. This refers to the ANC registration card used in the health centers. It is considered a “passport” for access to maternity wards, as well as care after delivery for the mother and newborn. Women perceive that those who do not seek ANC may be mistreated or penalized during delivery and the postpartum period.

*“Some women arrive at the clinic during the second or third month of pregnancy in order to “open the card”. [Nurse at a Health Center]*

Usually the first visit for ANC occurs during the second trimester of pregnancy. Women explained that this is often because they cannot receive services without pregnancy confirmation. Given that health centers do not have pregnancy tests available, confirmation of pregnancy is limited to visual or physical examination of a women’s belly. Primiparous women in the study area traditionally do not seek care until the pregnancy can be visually confirmed:

*"Depends… and sometimes varies, but it is not common for a two month pregnant woman to go to the clinic, they must first expect to receive advice at home. (…) According to our tradition we have to wait until the belly is visible, that confirms the pregnancy" [Focus Group with women at the community]*

*“When they arrive on the second month and we can´t confirm the pregnancy by the physical examination we request them to buy a test at a private pharmacy or come back when the pregnancy is visible. If I can´t feel the pregnancy we can´t open the file because I'm not sure if the woman is pregnant" [Nurse at a Health Center]*

There is a tradition that the first delivery should take place at home because it is the opportunity for the primiparous to learn from older and experienced women traditional knowledge regarding maternity. However we have observed that in general, women agree that it is important to give birth at maternities. They noted that women who did not attend ANC –and do not have the ANC registration card– would not dare to go for institutional delivery as they are afraid of being mistreated by the nurses. These women go to the health centers only for the postpartum visit.

#### External factors: transportation and economic factors

Lack of transportation and distance to facilities were recognized as barriers to seeking ANC by women and health care providers interviewed. Some health centers do not offer testing services, and must refer women to the nearest health facility where laboratory facilities are available. This creates an additional cost for women who must travel to another health center for testing. Women often, as a consequence, opt as an alternative to visit a traditional community midwife who is more accessible.

*Regarding anemia tests: "We used to have hemoglobinometers but they are broken since March (for more than 3 months at the moment of the interview). Now we send the women to the laboratory, they have the blood extraction there, however many times the results are not collected, it is not easy for the women to return to collect them because of the distance" [Health Center Official]*

Furthermore, the success of syphilis treatment ─which is free for pregnant women in Mozambique─ depends on families assuming the cost of treatment for partners in order to avoid reinfection.

## Discussion

This research shows that most of the ten components included in the ANC model are not fully implemented in the three study health centers. A number of contributing supply and demand side factors were identified, including factors related to organizational and structural issues, health care providers, and women’s perceptions of available care. The dysfunction of the public health sector supply chain is an urgent problem that must be prioritized for action since the implementation of the ANC model depends fundamentally upon the availability of a large package of specific supplies. Supply chain problems identified include poor coordination across and within levels of the health system; fragmentation of supply lines; lack of qualified and experienced staff; absence of clear protocols at each level; lack of reliable data for tracking and recording supplies; and deficiencies in transportation and health centers’ storage facilities.

Cultural beliefs coupled with the lack of pregnancy tests at health facilities influence women’s decision on when to seek ANC during pregnancy, which is often later on when the pregnancy becomes visible. Other studies also described that women hide pregnancy and delay prenatal care due to fear that they and their unborn infants would be targets of witchcraft or sorcery by jealous neighbors and kin [14]. Accessing ANC only after a pregnancy is visible reduces the number of ANC visits a woman can make prior to childbirth, and, consequently jeopardizes her ability to receive all recommended services. Delivery of effective tetanus and malaria preventive measures, for example, depends upon at least two visits to the health center, and testing and treatment of syphilis and HIV also depend upon a second appointment for follow-up treatment and counseling. Provision of folic acid and ferrous sulphate lacks effectiveness when administered later than the second trimester.

The most important reason women provided for attending ANC is to gain formal entrance into the healthcare system, including easier access to an institutional delivery and postpartum care. Similar to other studies in low-resource countries, our findings showed that women are not aware of the importance of each of the ANC interventions and do not, therefore, understand why professional care beyond one visit during pregnancy is critical to their health and the health of their unborn baby [14–16].

Also consistent with other research in low-resource settings, women identified lack of transportation, the distance to clinics, costs related to seeking care, inadequate infrastructure, and long waiting time at the health center as barriers to accessing ANC [17–20].

### Strengths and limitations of this study

To our knowledge this is the first qualitative analysis related to ANC undertaken in Mozambique with the full involvement of the Ministry of Health and with immediate application to the design and evaluation of an intervention to increase provider uptake of a package of evidence-based services for ANC [10].

Socio-demographic information of participants was not collected in this research, but each category of key informant participants was well described. Since the sessions were not audio-taped and the analysis were based on the notes taken by the interviewers, recall bias could be introduced.

Triangulation was performed to ensure validity of the information gathered. Key informants with different points of view were interviewed to try and capture as much information as possible from all stakeholders on factors impeding and facilitating uptake of the ANC model. Two analyst and at least two observers were involved in data analysis and review, and any discrepancies in coding or in interpretation of the findings were discussed until consensus was reached [21].

## Conclusions and recommendations

Based on the findings of this study, we suggest a number of interventions for improving the implementation of the ANC in Mozambique. These are described in Table 5 and suggest the need for profound and urgent revisions to the supply chain system, human resources in-practice training and supervision and environmental conditions. The next immediate steps for the team of experts conducting this research, including responsible individuals in the Ministry of Health, will be to use these results and conclusions to inform the development of an intervention to increase the use of a package of evidence-based services for ANC in Mozambique with the ultimate goal to improve maternal, perinatal and neonatal health outcomes [10].

**Table 5:** Recommendations to improve ANC in Mozambique

•	Strengthen delivery of care by (1) defining an integrated package that includes standardized tools for supervision; (2) adapting training models according to health care providers’ professional requirements, and (3) simplifying and updating protocols for ANC interventions.
•	Defining and strengthening a strategy for the organization of services delivery at the health facility level by addressing human resources efficiency while defining and developing a strategy for task shifting and realistically assessing the number of patients that can be attended by each provider.
•	Involve the community by disseminating information about the importance of each component of ANC, and pregnant women’s right to attend antenatal care for their health and the health of their unborn baby.
•	Regarding the supply chain for products required for the ANC interventions, we recommend to:
○	Introduce the use of an ANC kit system containing the necessary medicines, health commodities, laboratory supplies, equipment; provide adequate storage place for the kits that would give the provider easy and quick access to the necessary supplies.
○	Develop protocols for the procedures of obtaining and maintaining the stock of supplies required for implementation of the ANC package; introduce a tracking sheet to monitor the stock levels of the supplies.
○	Predefine a format for ordering products for the ANC package: use the number of ANC consultations as the basis for determining needed supplies.
○	Improve coordination and follow-up of medicines and health supplies by designating one person at the health center responsible for assessing the availability of the products necessary for ANC.

## Additional file

Additional file 1:
**Detailed methods.** Includes: population served by the clinics, detailed description of focus groups, detailed of the training conducted for the facilitators of the focus groups, description of the recording of the interviews and focus groups discussions, and the informed consent form. (PDF 92 kb)
