# Supplementary material for: Challenges and opportunities for implementing evidence-based antenatal care in Mozambique: a qualitative study
Source: BMC Pregnancy Childbirth. 2015 Sep 2;15:200. doi: 10.1186/s12884-015-0625-x (PMC4557743; doi:10.1186/s12884-015-0625-x)
Supplement: Additional file 1: — Detailed methods. Includes: population served by the clinics, detailed description of focus groups, detailed of the training conducted for the facilitators of the focus groups, description of the recording of the interviews and focus groups discussions, and the informed consent form. (PDF 92 kb) [file 12884_2015_625_MOESM1_ESM.pdf]

Additional file 1: Detailed methods

Population served by the clinics

This formative research served as basis to develop the intervention tested in the WHO Study A65709: Implementation of evidence-based antenatal care in Mozambique: a cluster randomized controlled trial. The Table below shows the selected ANC clinics in the WHO trial and the number of women attending 1st ANC visit in 2011. The three clinics included in this formative research are highlighted in red.

Region	Health Center (HC)	Women attending 1st ANC in 2011
**South**		
**Maputo City**	HC 1ro de Maio	3,165
**Maputo Province**	HC da Matola II	3,276
**Gaza**	HC annexed to the rural hospital of Chowke	3,480
**Gaza**	HC of Chibuto	9,756
**Central**		
**Sofala**	HC annexed to the district hospital of Dondo	2,728
**Tete**	HC annexed to the rural hospital of Songo	1281
**Tete City**	HC N° 2	2778
**North**		
**Nampula City**	HC 25 de Setembro	5,960
**Nampula District**	HC of Anchilo	1,894
**Cabo Delgado**	HC annexed to the rural hospital of Montepuez	4,182

Focus groups

The research protocol suggested 8-10 participants for each focus group. We had one group with women from the community with a size above these numbers (13 participants; three more than expected). We found that in this case women had high expectations and interest in this topic. We found that this was for them an opportunity to submit their concerns to people coming from outside. The researchers conducting the interview were aware and careful and the team had two facilitators (the local trained to facilitate in the local language and one of the main researcher) who assisted and supervised the quality of facilitation ensuring that difficulties raised were immediately corrected and safeguarded the correct conduct of the research protocol. Afterwards, researchers reported that the dynamics of the group were smooth with no bias or inequity in participation despite the relatively large size of the groups.

Training conducted for the facilitators of the focus groups

To select the facilitator we used the educational level criteria (minimum 10th class) combined with experience in data collection methods through in-depth individual interviews and conducting focus discussions, or experience in community animation (like activist).

The training intended to introduce the research assistants to the objectives of the research, data collection procedures, and tasks and responsibilities within the research team. The main focus was

to provide the necessary skills and study-specific knowledge related to the procedures and tools to be used in the fieldwork.

Main themes:

- Brief explanation of the qualitative formative research: nature, methodology, techniques, advantages and limitations;
- Interviews and focal group discussion: what it is, features and principles, how to facilitate, advantages and disadvantages. In this second topic the focus was on:
    - Procedures to facilitate focus group discussion;
    - The ethical issues such as: Free acceptance to be interviewed by obtaining informed consent; The importance of informing participants on the objectives of the study; protection of the identity of the participants; negotiating the best place and time to be interviewed;
    - How to pose questions for effective and respectful discussion: type of language to use; body language; Freedom to answer or not to answer; freedom to interrupt the interview if you wish; how to deal with sensitive and embarrassing questions; how to clarify in case of doubt; how to avoid inductive question; how to avoid questions about more ways than one in the same issue; how to avoid ambiguous words and expressions to pose questions;
- Taking notes
- Etc.

Recording of the interviews and focus groups discussions

The research envisioned the recording of the interviews. However, this was dependent upon the acceptance of the interviewees and the women in the focus groups. Regarding the interviews with the health professionals, because the topic was directly linked to their profession involving current work-related issues very pertinent in current Mozambique, none of the health professionals accepted to record the interview.

Women in the focus group discussions did not accept to be recorded either. Even if we clearly and unambiguously explained the objectives and circumstances of the research and they were reassured that the recordings and information would be treated confidentially and not be shared, the topics had to do with how women were treated at health centers and women did not trust where and how the recordings would be used. Women felt that if they accepted, they could not talk freely in the discussions and they would give politically correct answers instead of what they really wanted to say.

Informed consent

The informed consent is presented below. None of the women declined.

Projecto demonstrativo para a Implementação do Modelo da OMS sobre Cuidados Pré-Natais em Moçambique: Um Ensaio Clínico Aleatório Controlado

Consentimento Informado

Código do Participante ______________________________

Por ter sido convidado a participar no **Projecto demonstrativo para a Implementação do Modelo da OMS sobre Cuidados Pré-Natais em Moçambique: Um Ensaio Clínico Aleatório Controlado.**

Eu ……………………………………………………………………………….. declaro que:

1. Que aceito, de forma livre e sem ter sido sujeito a qualquer tipo de coação física e/ou moral, participar nesta Pesquisa.
2. Fui informado que o presente estudo tem por objectivo analisar as barreiras e factores facilitadores à implementação do pacote da OMS de cuidados pré-natais.
3. Fui devidamente esclarecido da natureza da minha participação neste estudo, dos riscos e benefícios que dela decorrem.
4. A minha participação no estudo consiste simplesmente em responder às questões que me forem colocadas de forma voluntária, podendo abandonar o estudo a qualquer momento se não desejar continuar.
5. Compreendi que não receberei nenhuma recompensa monetária por participar neste estudo
6. Compreendi que a informação relativa a minha participação será confidencial, e que em termos de grupo, a informação será utilizada pelo MISAU para encontrar a melhor estratégia para melhorar os cuidados pré-natais durante a gravidez.
7. Compreendi que caso haja dúvidas e demais esclarecimentos em relação a esta pesquisa, poderei contactar os investigadores, nos seguintes contactos: Adriano Biza (848697010) e Cornelia Catharina (Ingegorg) Jilles-Traas (843998511) ou ainda no Centro Internacional para a Saúde Reprodutiva, Rua José Macamo n.269, 1° andar, Tel: +25821328729/+258824797740 em Maputo
8. O meu consentimento para o efeito será validado pela minha assinatura e/ou impressão digital abaixo.

____ ______________

Assinatura do Participante Data

____ ______________

Assinatura da pessoa que obteve o consentimento Data

Projecto demonstrativo para a Implementação do Modelo da OMS sobre Cuidados Pré-Natais em Moçambique: Um Ensaio Clínico Aleatório Controlado

<u>Folha de Informação do Participante</u>

Bom dia (ou boa tarde),

O meu nome é <   >, e faço parte de um grupo de pesquisadores que está a fazer uma pesquisa que visa melhorar as intervenções incluídas no pacote de Cuidados Pré-Natais com o objectivo de melhorar a saúde da mãe e do seu bebe e para a qual gostaríamos de contar com a sua participação.

Para se saber como melhorar a saúde da mãe e do seu bebé, estamos a realizar esta pesquisa de carácter formativo, que colherá informação dos profissionais de saúde e das mulheres grávidas. Esta informação será obtida através de grupos focais e entrevistas.

Dada a importância que o seu testemunho (em tanto que trabalhador de saúde, mulher grávida utente ou não) tem para o sucesso do nosso trabalho, gostaríamos que você considerasse a possibilidade de participar nesta pesquisa, respondendo às perguntas que lhe vamos colocar, se não constituir algum inconveniente da sua parte.

Garantimos-lhe desde já que apesar de termos a sua assinatura na folha de consentimento, o grupo de pesquisa manterá confidencial, toda a informação que você prestar. A informação que nos dará será confidencial e de carácter privado, e que será usada apenas no âmbito do trabalho em causa. A equipa fará todos os esforços para proteger a sua minha identidade e nenhum/a participante será identificado/a em nenhum relatório ou publicação deste estudo ou os seus resultados.

Tem alguma pergunta quanto a informação que lhe foi dada? Deseja mais esclarecimentos sobre a finalidade da informação para melhor decidir sobre a sua participação?

É muito importante que falemos em privado, aonde é que acha que podemos fazer a entrevista?

O facto de participar na pesquisa não trará benefício directo para si em forma de pagamento de algum incentivo, mas servirá para ajudar a delinear e planificar actividades que melhorem a prestação de cuidados a mulher durante a gravidez.

Sinta-se livre de suspender a entrevista a qualquer momento.

Enquanto estiver a decorrer a pesquisa, os investigadores e os assistentes estarão disponíveis para esclarecer qualquer dúvida relacionada com a investigação.
